# Mycalamide A Shows Cytotoxic Properties and Prevents EGF-Induced Neoplastic Transformation through Inhibition of Nuclear Factors

**DOI:** 10.3390/md10061212

**Published:** 2012-05-30

**Authors:** Sergey A. Dyshlovoy, Sergey N. Fedorov, Anatoly I. Kalinovsky, Larisa K. Shubina, Carsten Bokemeyer, Valentin A. Stonik, Friedemann Honecker

**Affiliations:** 1 Laboratory of Marine Natural Products Chemistry, G.B. Elyakov Paciﬁc Institute of Bioorganic Chemistry, Far-East Branch of the Russian Academy of Sciences, Prospect 100-let Vladivostoku 159, Vladivostok 690022, Russia; Email: fedorov@piboc.dvo.ru (S.N.F.); kaaniv@piboc.dvo.ru (A.I.K.); shubina@piboc.dvo.ru (L.K.S.); stonik@piboc.dvo.ru (V.A.S.); 2 Department of Oncology, Haematology and Bone Marrow Transplantation with Section Pneumology, Hubertus Wald-Tumorzentrum, University Medical Center Hamburg-Eppendorf, Martinistr. 52, Hamburg 20246, Germany; Email: c.bokemeyer@uke.uni-hamburg.de (C.B.); f.honecker@uke.uni-hamburg.de (F.H.); 3 School of Natural Sciences, Far Eastern Federal University, Sukhanova Street, 8, Vladivostok 690091, Russia

**Keywords:** mycalamide A, *Polysincraton* sp., cancer preventive activity, apoptosis, AP-1, NF-κB, p53

## Abstract

Mycalamide A, a marine natural compound previously isolated from sponges, is known as a protein synthesis inhibitor with potent antitumor activity. However, the ability of this compound to prevent malignant transformation of cells has never been examined before. Here, for the first time, we report the isolation of mycalamide A from ascidian *Polysincraton* sp. as well as investigation of its cancer preventive properties. In murine JB6 Cl41 P^+^ cells, mycalamide A inhibited epidermal growth factor (EGF)-induced neoplastic transformation, and induced apoptosis at subnanomolar or nanomolar concentrations. The compound inhibited transcriptional activity of the oncogenic nuclear factors AP-1 and NF-κB, a potential mechanism of its cancer preventive properties. Induction of phosphorylation of the kinases MAPK p38, JNK, and ERK was also observed at high concentrations of mycalamide A. The drug shows promising potential for both cancer-prevention and cytotoxic therapy and should be further developed.

## Abbreviations

AP-1: activator protein-1
EGF: epidermal growth factor
FBS: fetal bovine serum
IC_50_: inhibition concentration 50%
INCC_50_: inhibition of number of colonies formed in soft agar concentration 50%
MAPK: mitogen activated protein kinases
MTS: 5-(3-carboxymethoxyphenyl)-2-(4,5-dimethylthiazolyl)-3-(4-sulfophenyl) tetrazolium, inner salt
NF-κB: nuclear factor kappa B
PI: propidium iodide

## 1. Introduction

Marine flora and fauna is a rich source of natural compounds that possess potent cancer preventive as well as cytotoxic activities [1,2,3,4,5,6,7,8]. We studied an ethanol extract of the ascidian *Polysincraton* sp. that was selected in a screening process due to its cytotoxic activity against the human cancer cell line HeLa. Cytotoxicity-guided fractionation of the extract resulted, among other findings, in the isolation of the previously described substance mycalamide A [9]. 

Mycalamides and related compounds are inhibitors of protein synthesis and show apoptosis-inducing activity [10,11]. Initially, they were isolated from the marine sponges *Mycale* sp. (mycalamide A, B and D) [12,13], *Stylinos* sp. (mycalamide C) [14], *Theonella* sp. (onnamides) and *Discodermia* sp. (theopederins), for review see [15]. Previously, mycalamide A was shown to be rather toxic, putting into question its potential as a cancer therapeutic [11,12,16,17]. However, cancer preventive activity of this compound at lower concentrations has so far not been examined. In the work presented here, we investigated the cancer preventive and pro-apoptotic properties of mycalamide A.

## 2. Results and Discussion

### 2.1. Isolation and Structural Identification of Mycalamide A from Ascidian *Polysincraton* sp.

The crude ethanolic extract of *Polysincraton* sp. possessed cytotoxic activity against the human cancer cell line HeLa, with an inhibitory concentration (IC_50_) < 62.5 μg/mL, determined by the MTS test [18]. Bioassay guided fractionation of the *Polysincraton* sp. extract led us to the isolation of the previously described mycalamide A. The substance was structurally identiﬁed by determination and comparison of its NMR and MS data, as well as physical constants with values published before [9,12].

Interestingly, we report isolation of mycalamide A from a representative of the subphylum Tunicata (family Didemnidae) for the first time. This finding strongly supports the hypothesis that symbiotic bacteria are the most likely origin of mycalamides and related compounds in marine invertebrates [19,20]. Surprisingly, extract of *Mycale* sp. has been reported to have an inhibitory effect on larvae settling of another ascidian, *Podoclavella moluccensis* [21]. The fact that we have isolated mycalamide A from the ascidian *Polysincraton* sp. suggests a species-specific character of this inhibition.

### 2.2. Mycalamide A Prevents EGF-Induced Transformation of JB6 Cl41 P+ Cells and Colony Growth of HeLa Cancer Cells

To assess whether mycalamide A exerts cancer preventive properties, we used EGF (10 ng/mL) as a promoter of neoplastic transformation of JB6 Cl41 P^+^ cells, a well established model of anchorage-independent growth in soft agar [22,23,24,25]. The JB6 cell system comprising clonal variants, including promotion sensitive (P^+^) and promotion resistant (P^−^) cells, or cells showing malignant transformation, is a valuable tool to identify compounds showing cancer preventive properties, and furthermore can be used to determine their action at the molecular level [26,27]. The JB6 P^+^, P^−^, and transformed variants are a series of cell lines representing early to late stages of neoplastic progression [22,23,28]. JB6 Cl41 P^+^ cells undergo neoplastic transformation upon stimulation with tumor promoters such as epidermal growth factor (EGF) or 12-*O*-tetradecanoylphorbol-13-acetate (TPA), resulting in anchorage-independent growth of colonies in soft agar. The transformation involves the activation of the nuclear factor AP-1, which regulates the transcription of various genes related to inflammation, proliferation, and metastasis [22,29,30]. Blocking AP-1- and NF-κB-transcriptional activity leads to inhibition of promoter-induced neoplastic transformation of these cells [22,31,32].

**Figure 1:**
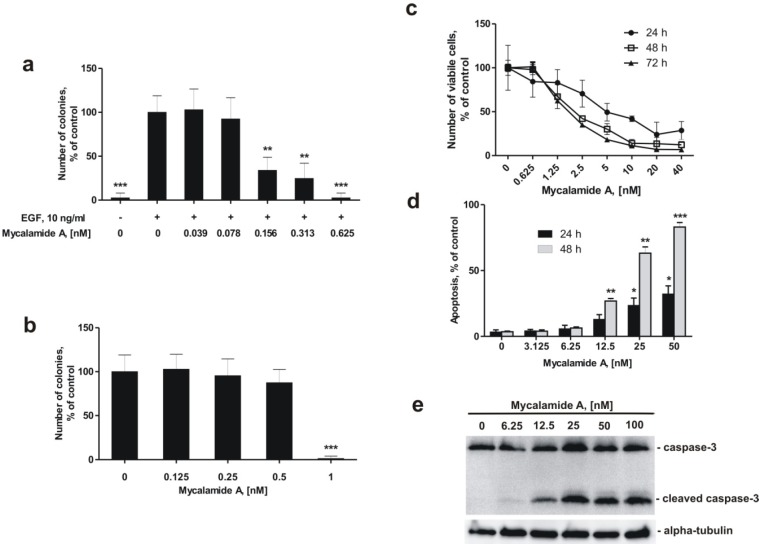
Effects of mycalamide A on cell viability, induction of apoptosis, neoplastic transformation, and colony formation. (**a**) Inhibition of EGF-induced neoplastic transformation of JB6 Cl41 P^+^ cells by mycalamide A; (**b**) Inhibition of colony formation of the human cancer cell line HeLa by mycalamide A; (**c**) Effect of mycalamide A on the proliferation of murine epidermal JB6 Cl41 P^+^ cells, analyzed by a cell proliferation assay; (**d**) Induction of apoptosis by mycalamide A in JB6 Cl41 P^+^ cells; (**e**) Caspase-3 cleavage in JB6 Cl41 P^+^cells treated with mycalamide A. All experiments were performed in triplicate. Statistically significant differences between treated and control cells are indicated as follows: * *p* < 0.05, ** *p* < 0.01, *** *p* < 0.005 (Student’s *t*-test).

As shown in Figure 1a, mycalamide A is able to inhibit EGF-induced neoplastic transformation of JB6 Cl41 P^+^ cells at very low, non-cytotoxic concentrations. The so called INCC_50_, the concentration leading to a 50% inhibition of colonies formed in soft agar, was 0.137 ± 0.035 nM after one week. This cancer preventive effect was observed at concentrations almost 50 times lower than the concentration causing 50% reduction (IC_50_) in the number of viable cells after 24 h treatment, which was determined at 6.32 ± 1.31 nM with the trypan blue exclusion method (Figure 1c).

Inhibition of colony growth by mycalamide A at non-cytotoxic concentrations was also observed in HeLa cells that show anchorage-independent growth in soft agar. The inhibition of colony formation of HeLa cells in soft agar was determined at an INCC_50_ = 0.67 nM (Figure 1b). This concentration was 8 fold lower than the IC_50_ of 5.5 ± 0.8 nM, determined in HeLa cells by the MTS test [18].

### 2.3. Mycalamide A Induces Apoptosis of JB6 Cl41 P^+^ Cells

Effects of mycalamide A on induction of apoptosis were determined by flow cytometry using double staining with annexin-V-FLUOS for binding of phosphatidylserine, and propidium iodide (PI) [33]. Although the number of viable JB6 Cl41 P^+^ cells decreased during the first 48 h of treatment with different concentrations of mycalamide A starting from 1.25 nM (Figure 1c), induction of apoptosis was not observed at concentrations below 6.25 nM (Figure 1d). This indicates that the decrease in cell numbers at low concentrations is caused by an inhibitory effect on cell growth rather than by induction of programmed cell death. Nonetheless, apoptosis was induced by mycalamide A in JB6 Cl41 P^+^ cells at concentrations of 12.5–50 nM (Figure 1d,e). We confirmed that mycalamide A induces apoptosis at higher concentrations by Western blotting. Exposing JB6 Cl41 P^+^ cells to mycalamide A concentrations of 12.5 nM and more for 24 h induced cleavage of caspase-3, a hallmark of apoptosis (Figure 1e).

### 2.4. Mycalamide A Inhibits AP-1-, NF-κB-, and p53-Dependent Transcriptional Activity in JB6 Cells

**Figure 2:**
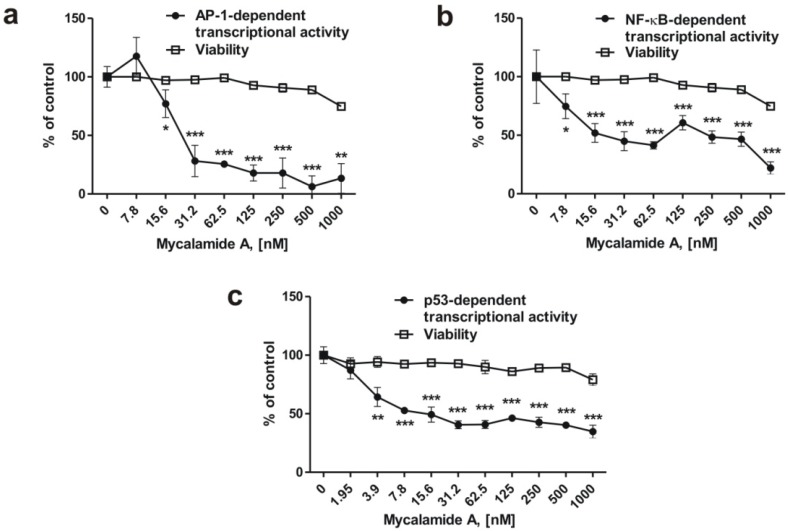
Inhibition of basal AP-1- (**a**), NF-κB- (**b**) or p53- (**c**) dependent transcriptional activity in JB6 Cl41 cells stably expressing a luciferase reporter gene controlled by AP-1, NF-κB, or p53 DNA binding sequences. All experiments were performed in triplicate. Statistically significant differences between treated and control cells are indicated as follows: * *p* < 0.05, ** *p* < 0.01, *** *p* < 0.005 (Student’s *t*-test).

It has previously been shown that inhibition of the transcriptional activity of the oncogenic nuclear factors AP-1 and NF-κB can result in cancer prevention [3,6,22]. We therefore examined the effects of mycalamide A on AP-1-, NF-κB-, and p53-dependent transcriptional activity using the JB6 Cl41 cell line stably expressing a luciferase reporter gene controlled by AP-1, NF-κB, or p53 DNA binding sequences, respectively. After 6 h of incubation with the indicated concentrations of mycalamide A, transcriptional activities of AP-1-, NF-κB-, and p53 were inhibited, while cell viability (determined with the MTS test) decreased only moderately, even at high concentrations (Figure 2a–c). The substance inhibited transcriptional activity of AP-1 or NF-κB after 6 h of treatment starting from concentrations of 7.8–15.6 nM (Figure 2a,b). Interestingly, in contrast to many pro-apoptotic drugs, mycalamide A did not increase p53-dependent transcriptional activity, although apoptosis was induced by the substance at that concentration (Figure 1d,e), suggesting that apoptosis induced by mycalamide A occurs via a p53-independent pathway. Similar findings have previously been reported for other cancer preventive agents, e.g., induction of cell cycle arrest and apoptosis by indole-3-carbinol or flavones from fruit and vegetable diet, without affecting p53 activity [34,35]. However, p53 is also known to induce apoptosis in a transcription-independent manner [36,37]. Therefore, further experiments are needed to clarify the role of p53 in mycalamide A-induced apoptosis.

### 2.5. Analysis of Phosphorylation of MAPK p38, JNK, and ERK under Mycalamide A Treatment

**Figure 3:**
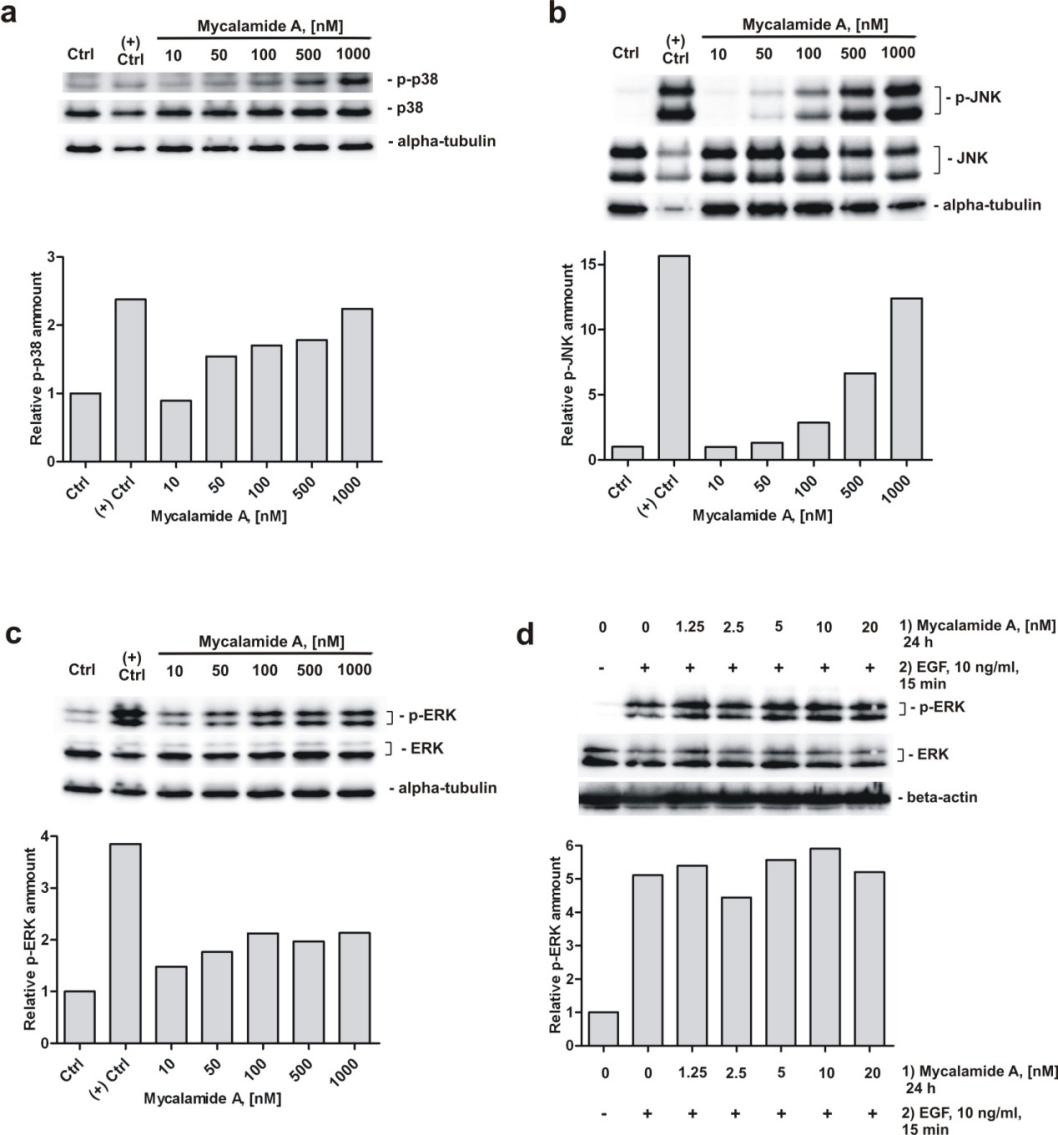
Analysis of MAPK p38, JNK, and ERK changes under mycalamide A treatment. Activation of p38 (**a**); JNK (**b**); and ERK (**c**) in JB6 Cl41 P^+^ cells treated with mycalamide A for 1 h. Anisomycin treated cells (50 μM for 1 h) were used as a positive control for detection of p38 and JNK phosphorylation, EGF-treated cells (10 ng/mL for 15 min) were used as positive control for detection of ERK phosphorylation; (**d**) Mycalamide A pretreatment does not inhibit EGF-induced ERK phosphorylation in JB6 Cl41 P^+^ cells. The relative amount of phosphorylated MAPK was quantified based on optical density of the signal intensity of the correspondent bands. The signal was normalized using beta-actin or alpha-tubulin. Primary and secondary antibodies used are listed in the supplementary data.

Furthermore, we investigated the role of several signaling pathways in the cellular response to mycalamide A. Western blotting analysis of JB6 Cl41 P^+^ cells treated with increasing concentrations of the substance for 1 h revealed a concentration-dependent induction of phosphorylation of p38, JNK and ERK (Figure 3a–c). Similar to mycalamide A, other marine alkaloids like polycarpine and its synthetic derivative dimethylpolycarpine, as well as marine sesquiterpenoid dactylone, have been reported to simultaneously activate p38, JNK and ERK kinases [38,39]. The ability to activate p38 kinase and JNK has previously been described for the structurally related compounds onnamide A and theopederin B, and it has been suggested that this activation is important for the antitumor activity of these compounds [40]. Our findings suggest that similar biological effects are induced by mycalamide A. For example, the activation of p38 and JNK could be involved in mycalamide A-induced apoptosis [41,42]. Therefore, we postulate that MAPK p38, JNK and ERK signaling pathways are involved in the cellular response to mycalamide A at least at high concentrations of the drug.

An important finding is our observation that pretreating JB6 Cl41 P^+^ cells with different concentrations of mycalamide A for 24 h in serum-free medium before EGF treatment (10 ng/mL) could not prevent EGF-induced phosphorylation of ERK (Figure 3d). It is conceivable that the cancer preventive effect might be associated with altered EGFR phosphorylation, leading to inhibition of the downstream signal transduction pathway. However, our result that mycalamide A is not able to prevent phosphorylation of ERK induced by EGF strongly suggests that the cancer preventive effect of mycalamide A is not mediated through direct or indirect inhibition of this pathway. However, this assumption awaits further experimental confirmation.

## 3. Experimental Section

### 3.1. Reagents and Antibodies

Mycalamide A was isolated and purified from the sea ascidian *Polysincraton* sp. as described below and was pure in accordance with NMR, MS, and TLC data. Anisomycin was purchased from Merk Chemicals (Nottingham, UK), epidermal growth factor (EGF) was purchased from Collaborative Research (Bedford, MA, USA), trypsin-EDTA solution and FBS were purchased from Invitrogen (Paisley, UK). The Cell Titer 96 Aqueous One Solution Reagent [3-(4,5-dimethylthiazol-2-yl)-5-(3-carboxymethoxyphenyl)-2-(4-sulfophenyl)-2*H*-tetrazolium, inner salt (MTS)] Kit was purchased from Promega (Madison, WI, USA). A list of the antibodies used is given in supplementary data.

### 3.2. Animal Material

The sea ascidian *Polysincraton* sp. was collected by scuba divers during the 36th scientific cruise of the research vessel “Akademik Oparin”, in August 2008, at 46°18′30′′N, 150°15′30′′E in the Natalyi Bay, off the Urup Island (Kuril Islands), Sea of Okhotsk, Russian Federation, at depths of 166–200 m. A voucher specimen is kept in the collection of the Pacific Institute of Bioorganic Chemistry, Vladivostok, Russia. Taxonomic identification was provided by Boris B. Grebnev. 

### 3.3. Isolation of Mycalamide A

Animal material (sea ascidian *Polysincraton* sp., 1000 g, wet weight) was frozen immediately after collection and later extracted with 2 L of EtOH. The ethanol extract, after evaporation *in vacuo*, was extracted with CHCl_3_ (2 × 200 mL). The chloroform extract was evaporated *in vacuo* (5 g) and subjected to separation on a silica gel column (150 × 40 mm). Fractions were subsequently eluted with 2000 mL of EtOAc/*n*-hexane (1:1) and 500 mL of CHCl_3_/MeOH (9:4). The CHCl_3_/MeOH fraction was evaporated *in vacuo* (260 mg) and subjected to separation on a YMC*GEL ODS-A column (90 × 25 mm) system using the solvent EtOH/H_2_O (7:3) as eluent. Fractions containing mycalamide A (TLC was used as control) were collected, evaporated in vacuo (47.2 mg) and separated with semi-preparative HPLC using Diasfer-110-C18 column and EtOH/H_2_O (1:1) as eluent. Mycalamide A was collected as a major peak of the chromatogram (5.2 mg, 0.00052% of animal wet weight).

### 3.4. Cell Culture

The JB6 Cl 41 P^+^ mouse epidermal cell line and it’s stable transfectants JB6-Luc AP-1, JB6-Luc NF-κB, and JB6-Luc p53 (PG-13) were cultured in monolayers at 37 °C and 5% CO_2_ in MEM, containing 5% FBS, 2 mM L-glutamine and 1% penicillin/streptomycin (Invitrogen, Paisley, UK). The human cancer cell line HeLa (originally cultured from a patient with a cervical carcinoma) was cultured at 37 °C and 5% CO_2_ in RPMI medium containing 10% FBS, 2 mM L-glutamine and 1% penicillin/streptomycin. JB6 cell lines were generously provided by Zigang Dong, Hormel Institute, University of Minnesota, MN, USA. The HeLa cell line was purchased from the ATCC collection. Information regarding the genetic background of these cell lines is available at the ATCC website.

### 3.5. Cell Proliferation Assay

Inhibition of cell growth of JB6 Cl41 P^+^ cells by mycalamide A was determined by the trypan blue exclusion method using the cell counter Vi-CELL Beckman Coulter (Krefeld, Germany). In brief, 8 × 10^4^ cells/well were seeded in 12-well plates and incubated overnight. The medium was replaced with fresh medium containing the indicated concentrations of mycalamide A in a total volume of 2 mL/well, and cells were incubated for 24, 48, and 72 h. Drug-containing medium was removed and cells were washed with 0.5 mL of PBS and trypsinized with 0.5 mL of trypsin-EDTA solution. The number of viable (trypan blue excluding) cells in both the medium and after trypsination was evaluated with the cell counter Vi-CELL according to the manufacture’s protocol. Assays were performed in triplicate.

### 3.6. Cytotoxicity Assay (MTS Test)

The effect of mycalamide A on HeLa cell viability was evaluated using the MTS test, using reduction of MTS into its formazan product [18]. The cells were pre-incubated overnight in 96-well plates (6 × 10^3^ cells/well) in medium, 100 μL/well. The medium was then replaced by fresh medium containing different concentrations of mycalamide A, and the cells were incubated for 22 h. Subsequently 20 μL of Cell Titer 96 Aqueous One Solution Reagent was added into each well, and MTS reduction was measured 2 h later spectrophotometrically at 492 and 690 nm as background using µQuant equipment (Bio-Tek Instruments, Winooski, VT, USA).

### 3.7. Detection of Apoptosis

Detection of induction of apoptosis by mycalamide A was performed by FACS-based analysis with Annexin-V-FLUOS (Roche, Mannheim, Germany) and propidium iodide (PI) (Sigma, Taufkirchen, Germany) double staining as previously described [33]. In brief, JB6 Cl41 P^+^ cells were pre-incubated overnight in 6-well plates (2 × 10^5^ cells/well). The medium was changed with fresh medium containing different concentrations of mycalamide A. After 48 h of treatment, cells were harvested with trypsin-EDTA solution, washed with PBS and incubated with 0.1 mL of Annexin-V-FLUOS and PI containing labelling buffer for 30 min in the dark at RT. Cells were analyzed by flow cytometry using a FACS Calibur apparatus (BD Bioscience, Bedford, MA, USA). The results were analyzed with the Cell Quest Pro software (BD Bioscience, Bedford, MA, USA).

### 3.8. Anchorage-Independent Neoplastic Transformation or Colony Growth Assay

The cancer preventive effect of mycalamide A was evaluated using an anchorage-independent neoplastic transformation assay. Briefly, EGF (10 ng/mL) was used to induce neoplastic transformation of JB6 Cl41 P^+^ cells. The assay was carried out in 6-well tissue culture plates. Mouse JB6 Cl41 P^+^ cells (8 × 10^3^ cells/mL) were treated with various concentrations of mycalamide A in 1 mL of 0.33% basal medium Eagle (BME)-agar containing 10% FBS over 3 mL of 0.5% BME-agar containing 10% FBS and various concentrations of mycalamide A. The cultures were maintained at 37 °C, 5% CO_2_ for a time period of 1 week, after which the number of cell colonies was scored using an Olympus CKX31 inverted research microscope (Olympus, Center Valley, PA, USA). The ability of mycalamide A to inhibit the growth of cell colonies of the human cancer cell line HeLa in soft agar was determined using the same protocol, however without EGF-stimulation.

### 3.9. Protein Preparation and Western Blotting

Preparation of protein extracts for Western blotting was performed as described previously [43,44,45]. In brief, 1 × 10^6^ cells/well were seeded in Petri dishes and incubated overnight. The medium was replaced with fresh medium containing substance at the indicated concentrations in a total volume of 10 mL/dish, and cells were incubated for the indicated times. Cells were harvested by mechanical scratching, pelleted by centrifugation for 5 min at 453× g, and washed 3 times with PBS, followed by pelleting using the same conditions. Cells were lysed with 100 µL of lysis buffer (0.88% [w/v] NaCl, 50 mM Tris-HCl (pH 7.6), 1% NP-40 [v/v], 0.25% [w/v] NaClO_2_, 1 mM PMSF, 1 mM Na_3_VO_4_) on ice for 20 min. Lysates were frozen overnight at −20 °C and centrifuged 10 min at 11,170× g. Protein concentration in the supernatants was determined using the Bradford assay. Protein extracts were diluted with lysis buffer and loading dye up to a total protein concentration of 1 µg/µL, heated 5 min at 99 °C, and subjected to electrophoresis in 15% SDS-PAGE at 120 V. Proteins were transferred from gel to a 0.2 µm pore PVDF membrane (Millipore, Bedford, MA, USA) at 20 V for 1 h. The membrane was blocked with 5% [w/v] non-fat dry milk in 0.05% Tween-20/TBS before treatment with the primary antibodies, according to the manufacturer’s protocol. After washing, the membrane was incubated with the appropriate secondary antibody during 1 h at RT. Signals were detected using the ECL chemiluminescence system (Thermo Scientific, Rockford, IL, USA) according to the manufacturer’s protocol. Relative optical density of the signal intensity of the bends was quantified with Quantity One 4.6 software (BioRad, Hercules, CA, USA).

### 3.10. Determination of the Effect of Mycalamide A on the Basal Transcriptional Activity of AP-1, NF-κB or p53

The effect of mycalamide A on the basal transcriptional activities of AP-1, NF-κB, or p53 was evaluated using JB6 Cl41 cell lines stably expressing a luciferase reporter gene controlled by an AP-1-, NF-κB-, or p53-DNA binding sequence, as described previously [6]. Briefly, cells were pre-incubated overnight in 96-well plates (6 × 10^3^ cells/well) in culture medium (100 μL/well). Then the medium was replaced with fresh medium containing different concentrations of mycalamide A. After incubation for 6 h, cells were lysed for 1 h at RT with lysis buffer (0.1 M PBS (pH 7.8), 1% Triton X-100, 1 mM DTT, 2 mM EDTA). Then, 30 μL of lysate from each well was transferred into a plate for luminescent analysis, and luciferase activity was measured using luciferase assay buffer (100 μL/well) (0.47 mM D-luciferin, 20 mM Tricin, 1.07 mM (MgCO_3_)_4_ × Mg(OH)_2_ × 5H_2_O, 2.67 mM MgSO_4_ × 7H_2_O, 33.3 mM DTT, 0.53 mM ATP, 0.27 mM CoA, and 0.1 mM EDTA (pH 7.8)) and the Luminoscan Ascent Type 392 microplate reader (Labsystems, Helsinki, Finland).

## 4. Conclusions

Mycalamide A shows promising potential for both cancer prevention and cytotoxic therapy, warranting further exploration. Inhibition of EGF-induced neoplastic transformation by mycalamide A can be explained, at least in part, by downregulation of AP-1- and NF-κB-dependent transcriptional activity. At higher concentrations, the substance induces apoptosis which is executed via caspase-3. Furthermore, the kinase signaling pathways MAPK p38, JNK, and ERK are involved in the cellular response to mycalamide A. These results help to better understand the antitumor activity of this interesting marine natural compound. 

## Supplementary Files

Supplementary File 1: PDF-Document (PDF, 695 KB)
