# Supplementary material for: Mycalamide A Shows Cytotoxic Properties and Prevents EGF-Induced Neoplastic Transformation through Inhibition of Nuclear Factors
Source: Mar Drugs. 2012 May 30;10(6):1212–24. doi: 10.3390/md10061212 (PMC3397435; doi:10.3390/md10061212)

Supplementary Materials

Table 1. Description of aantibodies
Figure 1. Effect of mycalamide A on cell viability of HeLa cells, detected by the cytotoxicity assay (MTS test)
Figure 2A. ^{1}H NMR spectrum of mycalamide A in CD_3OD (0–6.0 ppm)
Figure 2B. ^{1}H NMR spectrum of mycalamide A in CD_3OD (crop 0–1.8 ppm)
Figure 2C. ^{1}H NMR spectrum of mycalamide A in CD_3OD (crop 1.8–2.7 ppm)
Figure 2D. ^{1}H NMR spectrum of mycalamide A in CD_3OD (crop 3.0–4.0 ppm)
Figure 2E. ^{1}H NMR spectrum of mycalamide A in CD_3OD (crop 4.0–4.9 ppm)
Figure 2F. ^{1}H NMR spectrum of mycalamide A in CD_3OD (crop 5.0–6.0 ppm)
Figure 3. ^{13}C NMR spectrum of mycalamide A in in DMSO-d_6

Table 1. Description of antibodies.

Antibodies	Clonality	Source	Cat.-No.	Dilution Used	Manufacturer
anti-α-Tubulin	mAb	mouse	T5168	1:5000	Sigma-Aldrich
anti-β-actin	mAb	mouse	CP01	1:10000	Calbiochem
anti-caspase-3	mAb	rabbit	#9665	1:1000	Cell Signaling
anti-ERK	mAb	mouse	#9107	1:2000	Cell Signaling
anti-JNK	mAb	rabbit	#9258	1:1000	Cell Signaling
anti-p38	mAb	rabbit	#9212	1:1000	Cell Signaling
anti-phospho-ERK	mAb	rabbit	#4377	1:1000	Cell Signaling
anti-phospho-JNK	mAb	rabbit	#4668	1:1000	Cell Signaling
anti-phospho-p38	mAb	rabbit	#4511	1:1000	Cell Signaling
anti-mouse IgG-HRP		sheep	NXA931	1:10000	GE Healthcare
anti-rabbit IgG-HRP		goat	#7074	1:5000	Cell Signaling

Figure 1. Cell viability of HeLa cells under treatment with different concentrations of mycalamide A. Cytotoxicity was determined using the MTS method. Statistically significant differences (determined by *t*-test) between treated and control cells are indicated as follows: ** p <0.01, *** p <0.005.

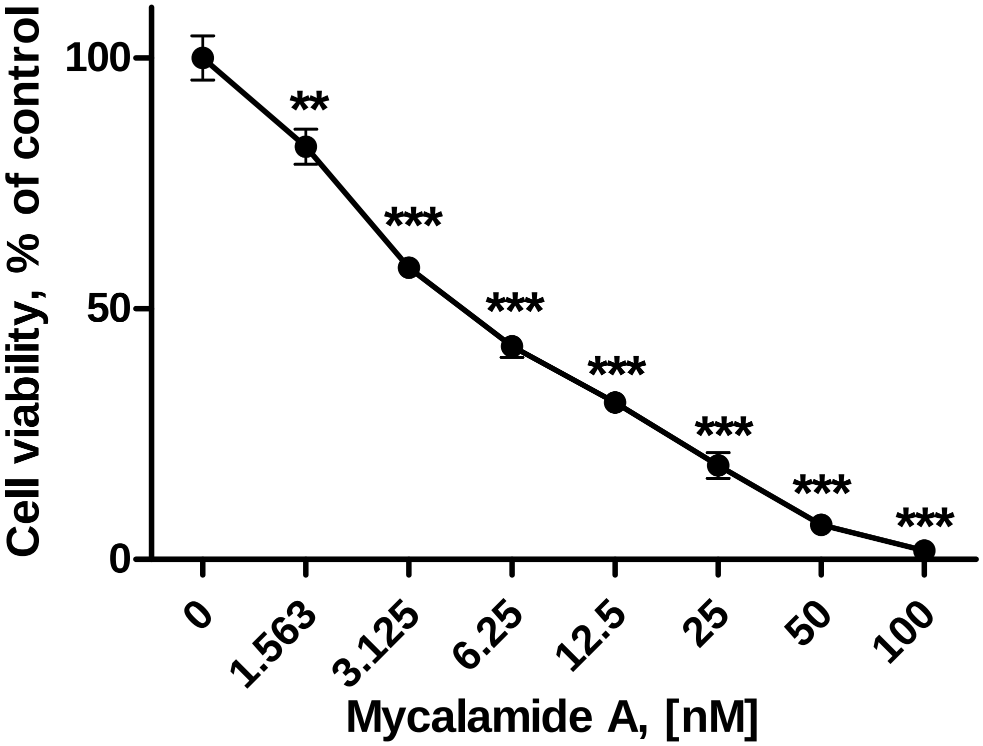

Figure 2A. ^{1}H NMR spectrum of mycalamide A in CD_3OD (0–6.0 ppm).

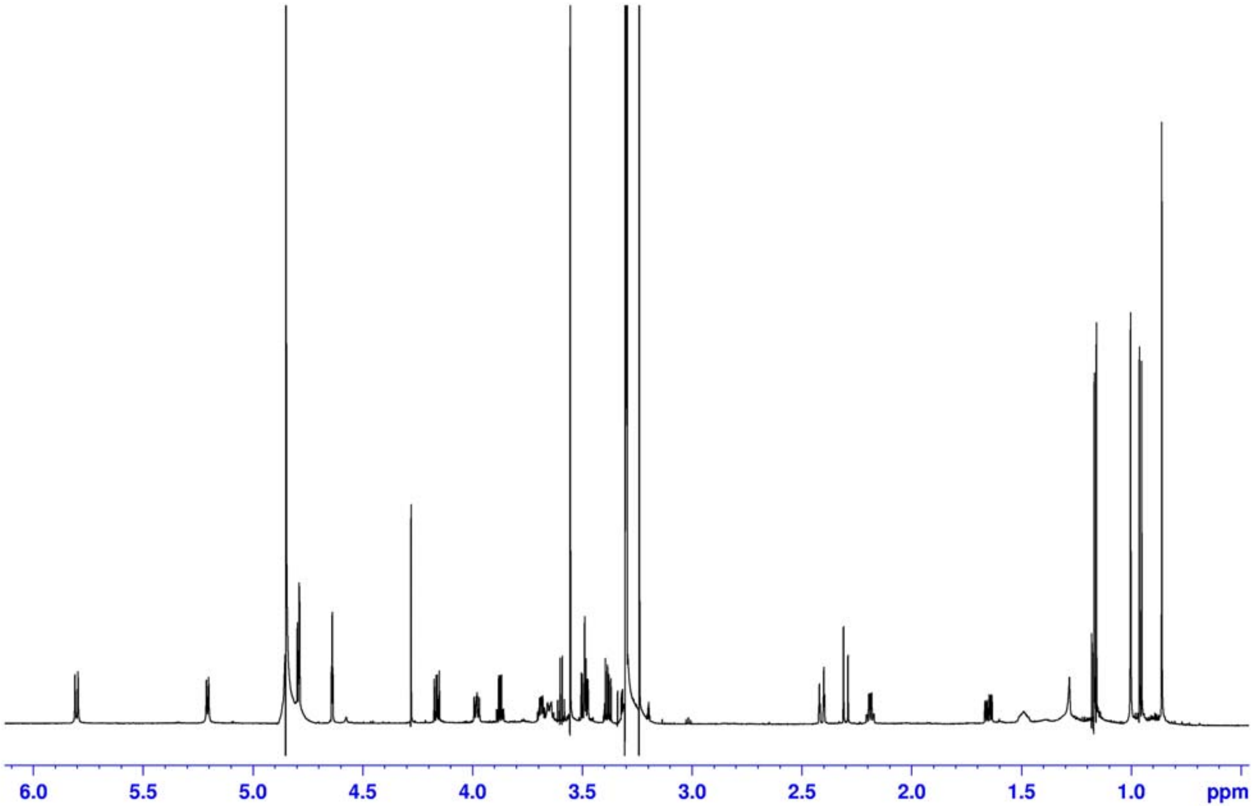

Figure 2B. ^{1}H NMR spectrum of mycalamide A in CD_3OD (crop 0–1.8 ppm).

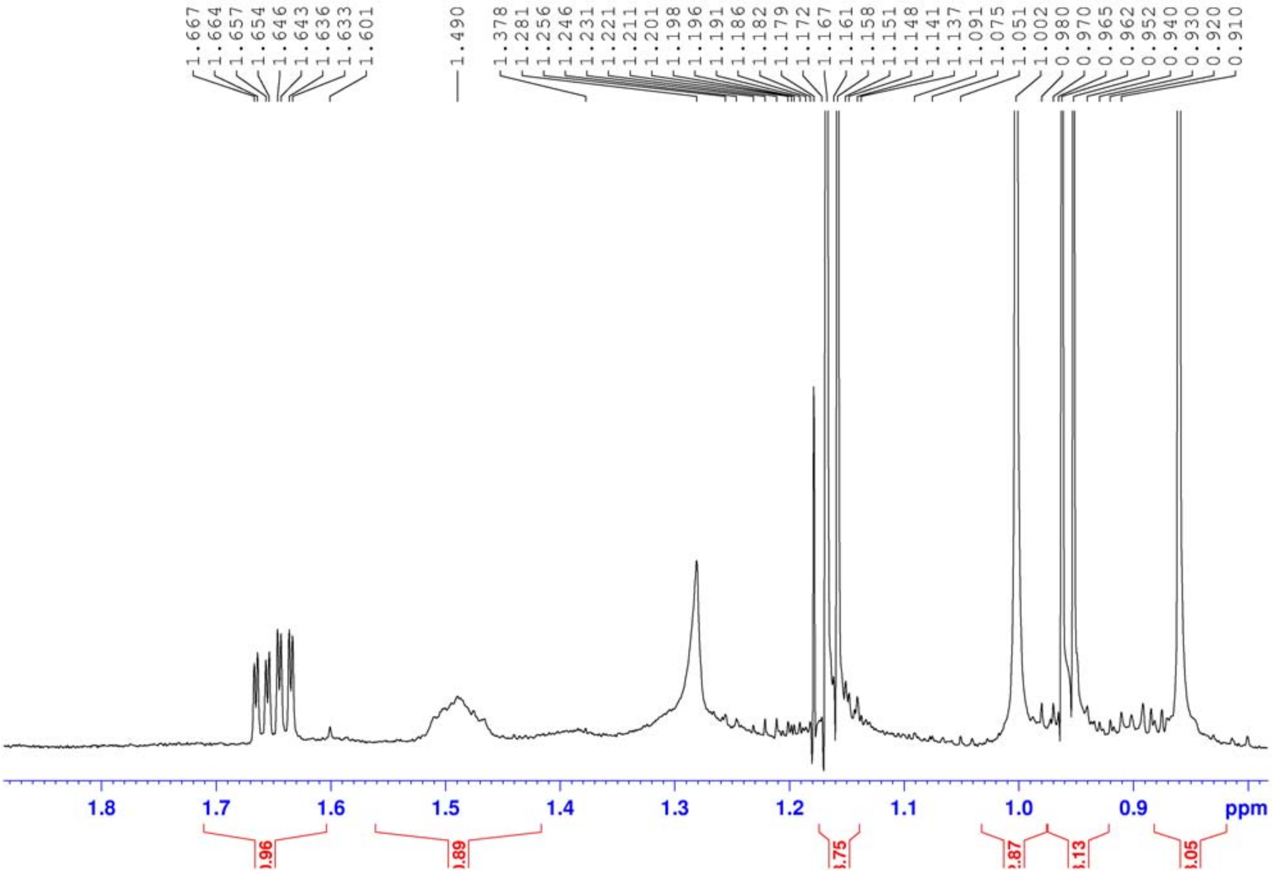

Figure 2C. ^{1}H NMR spectrum of mycalamide A in CD_3OD (crop 1.8–2.7 ppm).

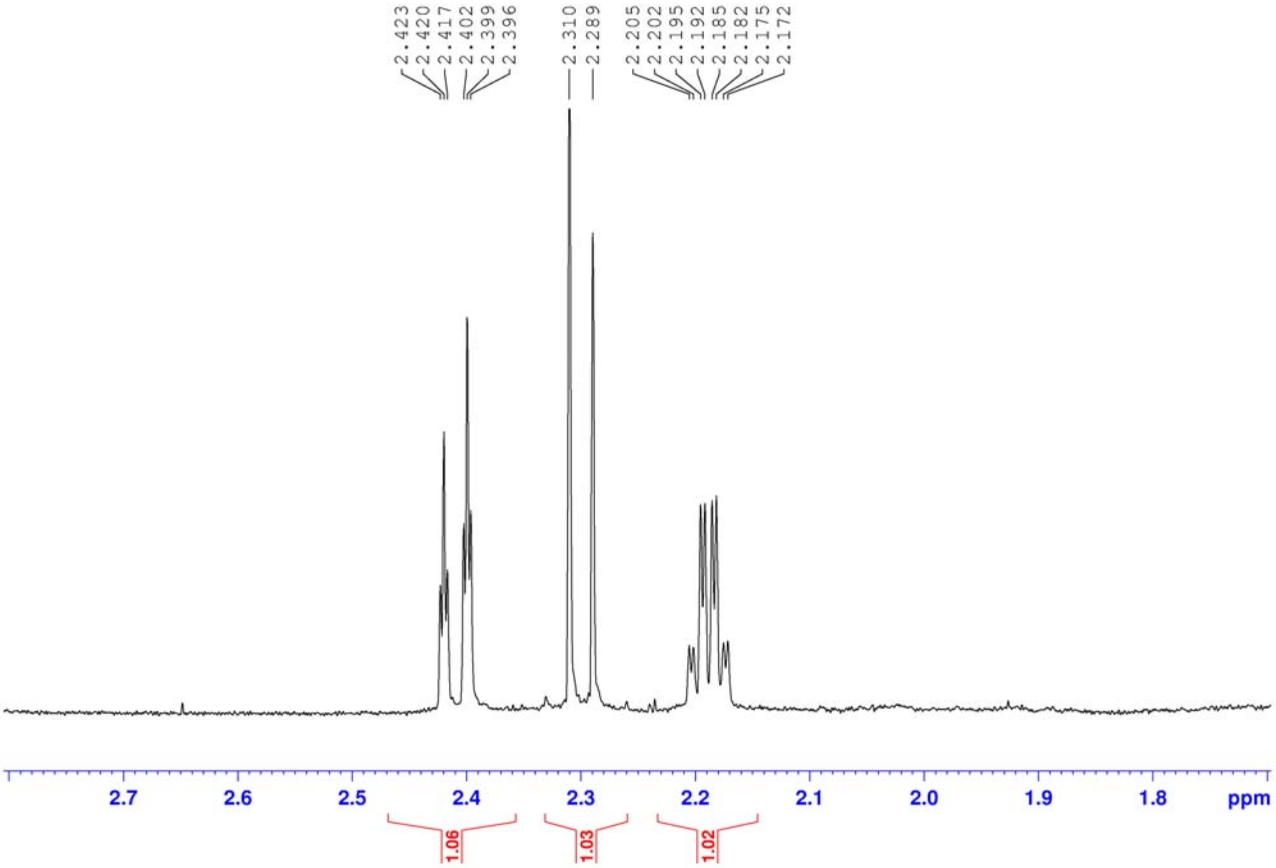

Figure 2D. ^{1}H NMR spectrum of mycalamide A in CD_3OD (crop 3.0–4.0 ppm).

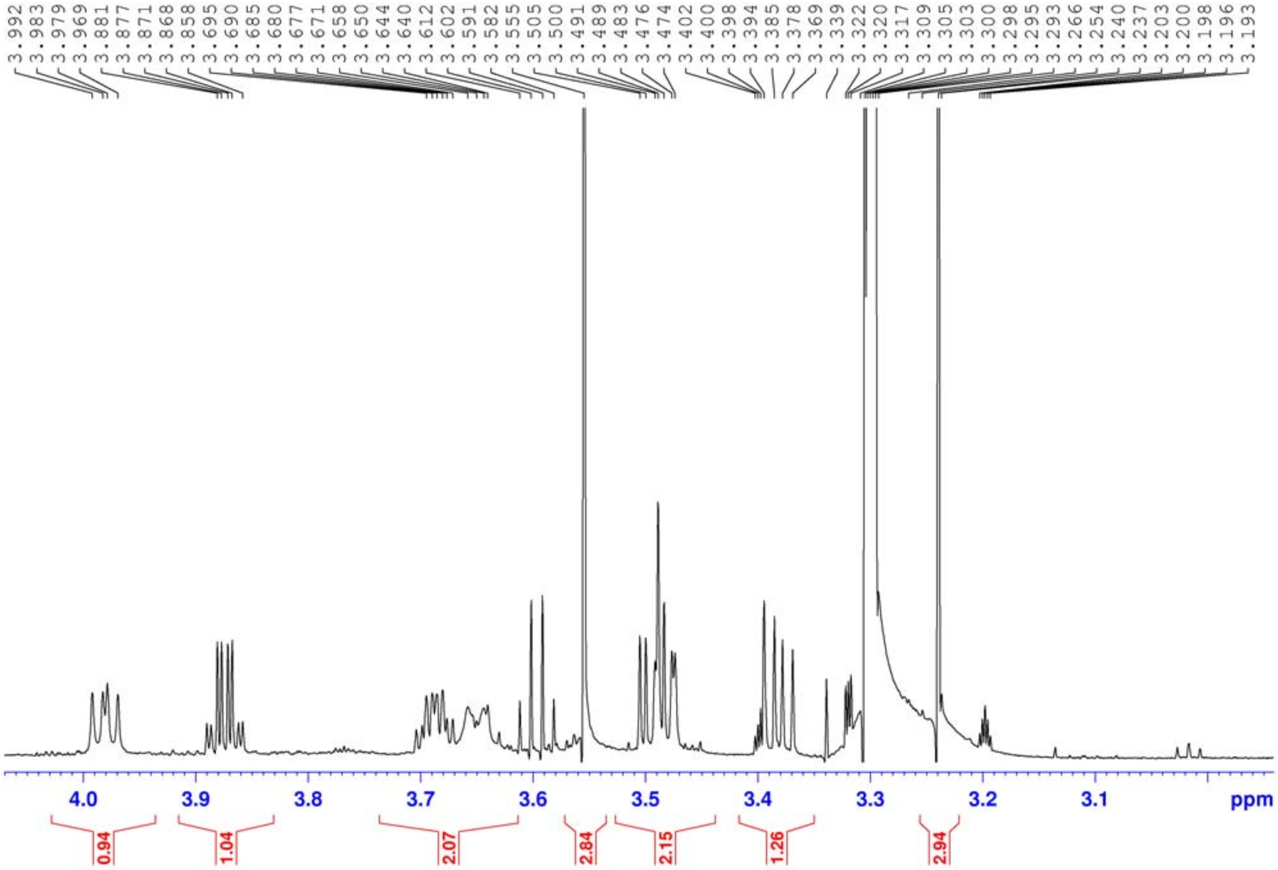

Figure 2E. ^{1}H NMR spectrum of mycalamide A in CD_3OD (crop 4.0–4.9 ppm).

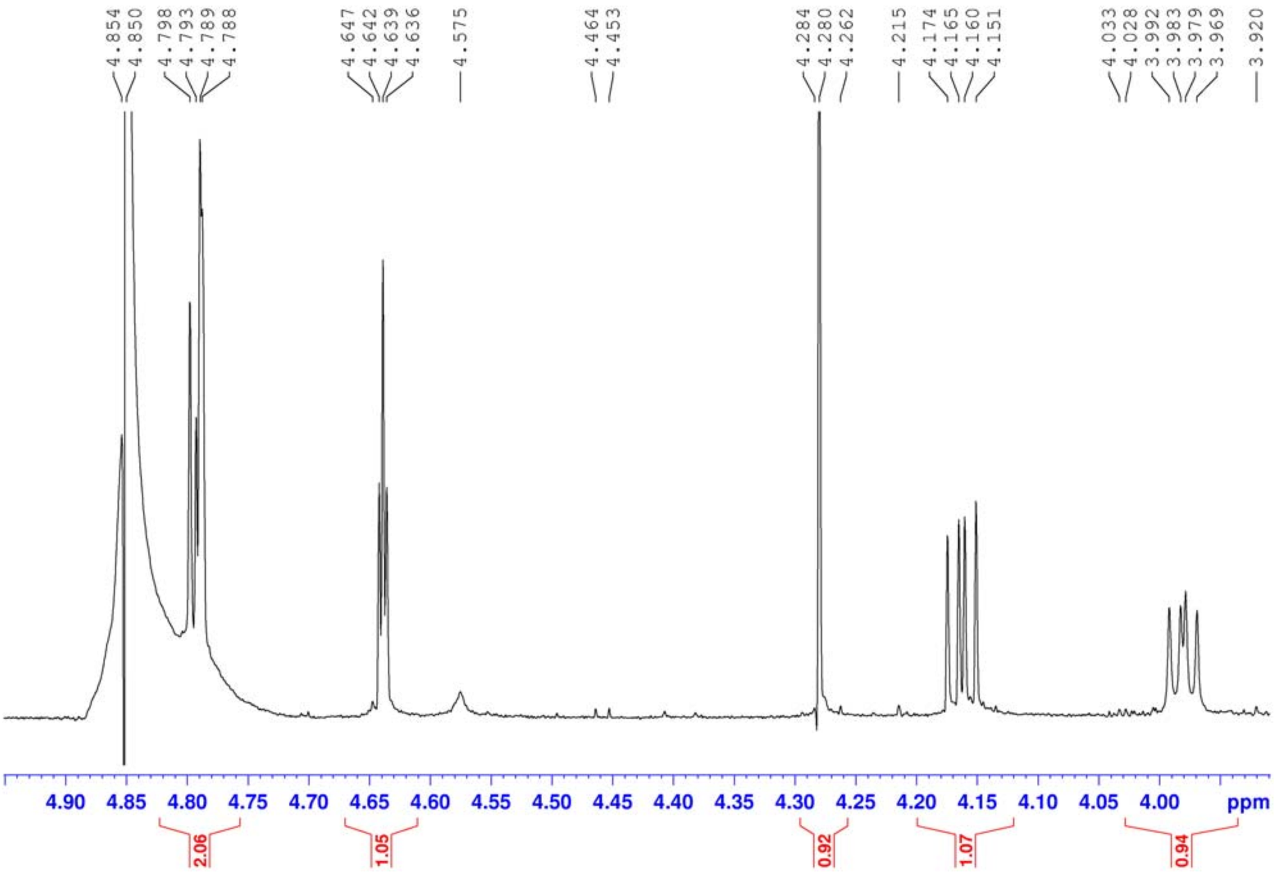

Figure 2F. ^{1}H NMR spectrum of mycalamide A in CD_3OD (crop 5.0–6.0 ppm).

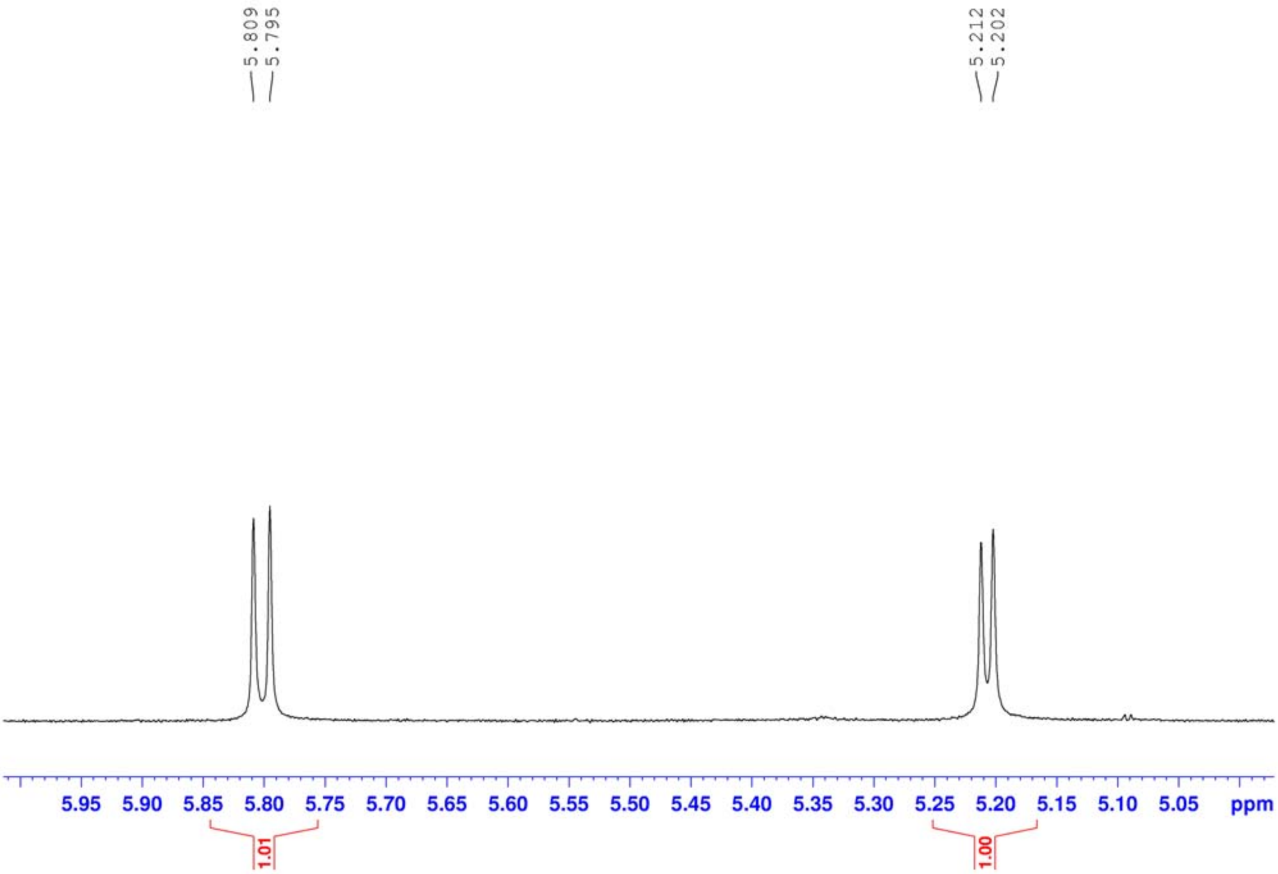

Figure 3. ^{13}C NMR spectrum of mycalamide A in DMSO-d_6.

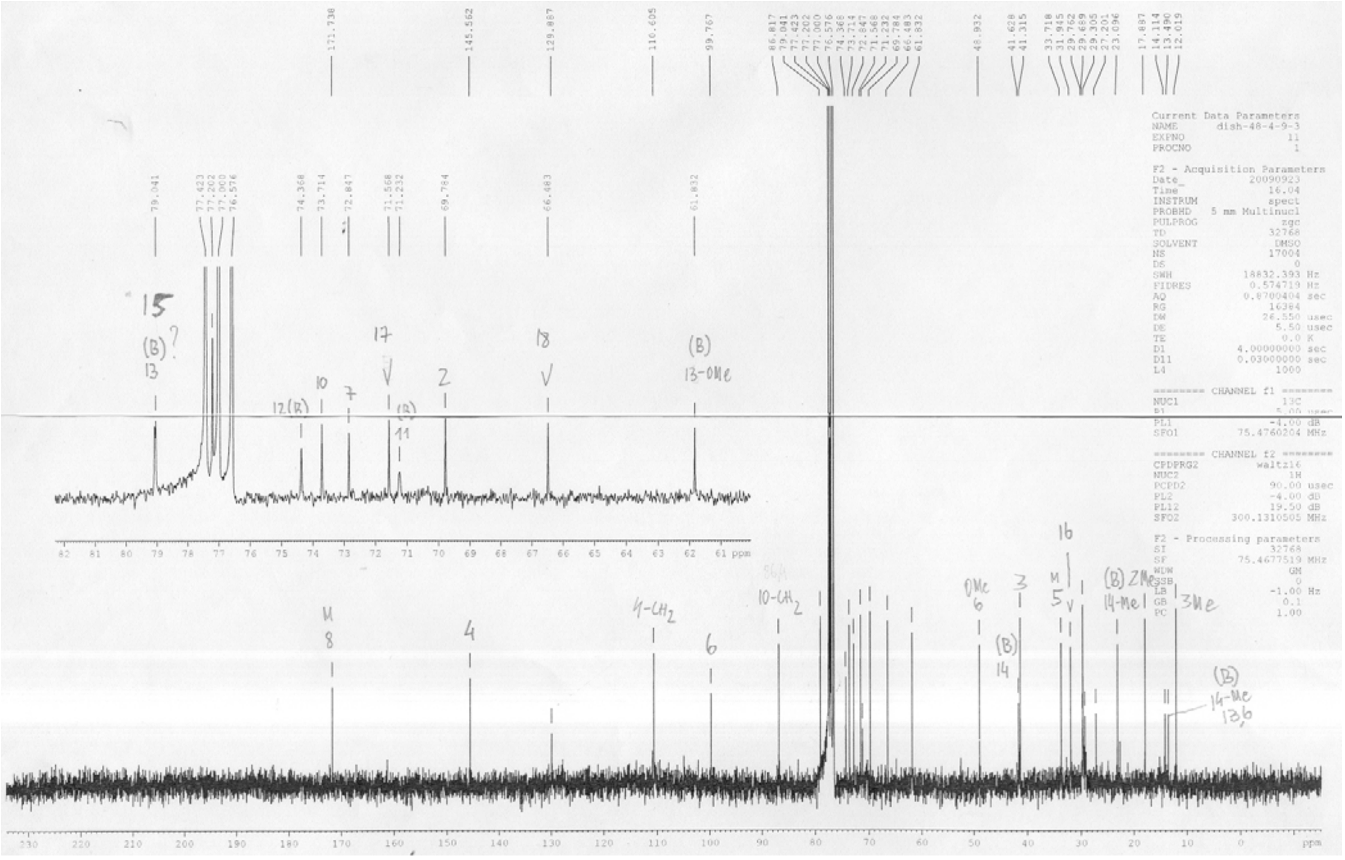

Supplement: Supplementary File 1: — PDF-Document (PDF, 695 KB) [file marinedrugs-10-01212-s001.pdf]
